# *AutoDensity*: an automated method to measure mammographic breast density that predicts breast cancer risk and screening outcomes

**DOI:** 10.1186/bcr3474

**Published:** 2013-09-11

**Authors:** Carolyn Nickson, Yulia Arzhaeva, Zoe Aitken, Tarek Elgindy, Mitchell Buckley, Min Li, Dallas R English, Anne M Kavanagh

**Affiliations:** 1 Centre for Women’s Health, Gender and Society, Melbourne School of Population and Global Health, University of Melbourne, 207 Bouverie Street, Melbourne, VIC 3010, Australia; 2 Centre for Mathematics, Informatics and Statistics, Commonwealth Scientific and Industrial Research Organisation, 5 Julius Avenue, North Ryde, Sydney, NSW 2113, Australia; 3 Department of Computing, Macquarie University, E6A Eastern Rd, Sydney, NSW 2113, Australia; 4 Centre for MEGA Epidemiology, Melbourne School of Population and Global Health, University of Melbourne, 207 Bouverie Street, Melbourne, VIC 3010, Australia; 5 Cancer Epidemiology Centre, Cancer Council Victoria, 100 Drummond Street, Melbourne, VIC 3053, Australia

## Abstract

**Introduction:**

While *Cumulus* – a semi-automated method for measuring breast density – is utilised extensively in research, it is labour-intensive and unsuitable for screening programmes that require an efficient and valid measure on which to base screening recommendations. We develop an automated method to measure breast density (*AutoDensity*) and compare it to *Cumulus* in terms of association with breast cancer risk and breast cancer screening outcomes.

**Methods:**

*AutoDensity* automatically identifies the breast area in the mammogram and classifies breast density in a similar way to *Cumulus*, through a fast, stand-alone Windows or Linux program. Our sample comprised 985 women with screen-detected cancers, 367 women with interval cancers and 4,975 controls (women who did not have cancer), sampled from first and subsequent screening rounds of a film mammography screening programme. To test the validity of *AutoDensity*, we compared the effect estimates using *AutoDensity* with those using *Cumulus* from logistic regression models that tested the association between breast density and breast cancer risk, risk of small and large screen-detected cancers and interval cancers, and screening programme sensitivity (the proportion of cancers that are screen-detected). As a secondary analysis, we report on correlation between *AutoDensity* and *Cumulus* measures.

**Results:**

*AutoDensity* performed similarly to *Cumulus* in all associations tested. For example, using *AutoDensity*, the odds ratios for women in the highest decile of breast density compared to women in the lowest quintile for invasive breast cancer, interval cancers, large and small screen-detected cancers were 3.2 (95% CI 2.5 to 4.1), 4.7 (95% CI 3.0 to 7.4), 6.4 (95% CI 3.7 to 11.1) and 2.2 (95% CI 1.6 to 3.0) respectively. For *Cumulus* the corresponding odds ratios were: 2.4 (95% CI 1.9 to 3.1), 4.1 (95% CI 2.6 to 6.3), 6.6 (95% CI 3.7 to 11.7) and 1.3 (95% CI 0.9 to 1.8). Correlation between *Cumulus* and *AutoDensity* measures was 0.63 (*P* < 0.001).

**Conclusions:**

Based on the similarity of the effect estimates for *AutoDensity* and *Cumulu*s in models of breast density and breast cancer risk and screening outcomes, we conclude that *AutoDensity* is a valid automated method for measuring breast density from digitised film mammograms.

## Introduction

Population mammographic screening might be more effective if screening strategies were tailored according to mammographic breast density [1,2]. Women with high breast density are at higher risk of breast cancer [3] and in population mammographic breast cancer screening programmes they experience reduced screening programme sensitivity (the proportion of cancers that are screen-detected) [4,5] and larger tumours at diagnosis [5,6]. This is due to increased cancer risk and because dense areas on mammograms can obscure cancers [4-9].

Specific strategies for tailoring screening according to breast density include adding ultrasound to mammography [10-12], using magnetic resonance imaging (MRI) instead of mammography [13], or more frequent screening of women with high breast density potentially offset by less frequent screening of women with low breast density [2,14]. Trials of MRI or adjunctive ultrasound for women with dense breasts suggest improvements in cancer detection but increases in false positive rates [10,15].

Current methods of measuring breast density are not suitable for developing tailored screening strategies because they are manual methods that are time-consuming and have inadequate reliability. In the USA, radiologists routinely use the *BI-RADS™* (Breast Imaging Reporting and Data System) visually estimated categories [16], however, this method has limited within-reader reliability (κ = 0.71) and between-reader reliability (κ = 0.54) [17], and it is not sufficiently discriminatory because it has only four categories of breast density and a majority of women (for example 81% [18], or 92% [19]) are classified in the middle two categories.

A widely used computer-assisted method is *Cumulus*[20]. It measures breast density on a continuous scale and has high between- and within-reader reliability in carefully monitored research settings [21], however, this may not be reliably realised in the routine screening context, and the method is time-consuming and costly in terms of staff time.

If breast density is to be routinely measured in screening programmes, an efficient and high-quality automated method is required. Ideally, trials of alternate screening strategies should be based on such a method so that the evidence can be easily translated into screening programmes.

Several automated measurement methods have recently been published [22-31]. All these methods offer perfect inter-read reliability. Some methods segment the distinctly white tissue (essentially automating the *Cumulus* approach) [25,26] while others estimate the underlying volumes of dense and non-dense breast tissue by projecting two-dimensional information onto three-dimensional space [27-30]. Some automated methods have shown associations with breast cancer risk comparable to *Cumulus* and *BI-RADS™* measures [25,27] but none have yet been validated against important measures of the effectiveness of screening programmes such as programme sensitivity, interval cancer rates and tumour size at diagnosis. Validation of methods against screening outcomes is required because the way that breast density affects breast cancer risk is likely to differ from the way that it affects radiological reading of mammograms.

In this study, we describe an efficient, automated method for measuring breast density and compare it with *Cumulus* in terms of predicting breast cancer risk, screening programme sensitivity, risk of small and large screen-detected cancers and interval cancers, and tumour size at diagnosis. We utilise an existing study database of BreastScreen Australia film mammograms from screening episodes and their associated *Cumulus* breast density measurements and clinical and screening data [21,32,33].

## Methods

### Participants

BreastScreen Australia is a free population-based breast screening programme that offers biennial screening to women aged 40 years and above, specifically targeting women aged 50 to 69 years. For this study, the source population comprised all women who attended the BreastScreen Australia programme in the state of Victoria (BreastScreen Victoria), either for first round screening in 1994 or 1995 (and who reported no previous mammogram), or subsequent round screening in 1995 or 1996 (excluding women in the first round sample). Women were eligible for the study if they were 79 years or younger, had no self-reported history of breast cancer and no ‘significant’ breast cancer symptoms at the time of screening (breast lump not examined by a doctor or a blood-stained or watery nipple discharge). We included only women with a *Cumulus* breast density reading from our previous studies. Our database included updated BreastScreen Victoria data received in October 2005, which resulted in minor changes in the sample available for this analysis compared to our previous publications [21,32,33].

Selection of participants followed methods used previously [33], where cases were all eligible women with invasive screen-detected or interval breast cancers (675 screen-detected and 183 interval cancer cases at first round screening and 344 screen-detected and 198 interval cancer cases at subsequent rounds), women diagnosed with ductal carcinoma *in situ* (DCIS) were not included in the study, and controls consisted of a sample of screened women with no cancer diagnosis (either true-negative or false-positive screening outcomes) selected randomly from both first and subsequent screening rounds (2,051 women from first round screening and 3,267 women from subsequent rounds, corresponding to sampling fractions of 1.55% and 3.25% respectively).

### Cancer ascertainment and classification

Screen-detected cancers were recorded by BreastScreen Victoria and interval cancers were identified by linking the population-based Victorian Cancer Registry to BreastScreen Victoria records, providing near-complete ascertainment of interval cancers [21,34]. Tumour size was recorded as the widest cross-section of the largest lesion as reported in the pathology report. Consistent with national protocols, tumours coded as microinvasive were assigned a size of 0.1 mm and screen-detected cancers were categorised as small (≤15 mm) or large (>15 mm) [34].

### Questionnaire data

Participants completed a questionnaire at the time of their screening appointment that included questions on family history of breast cancer (a first-degree relative versus no first-degree relative), current hormone therapy use (yes/no), country of birth and symptoms (none, or no ‘significant’ symptoms defined as any breast symptoms other than a breast lump or blood-stained or watery nipple discharge).

### Digitised mammograms

Mammograms were originally taken on a range of analogue (film) mammography machines. Cranio-caudal (CC) views were scanned in the late 1990s using a single digitiser (digitiser specifications and settings not known). Each scanned image included various background artefacts such as tags indicating breast laterality and radiological view, nameplates, and bright borders generated during scanning.

### ***Cumulus*** measurements

*Cumulus* was used previously to measure breast density from the digitised images, with measurements available for 93% of available cases included in previous analyses [24,33]. We used measurements from the cancer-affected breast where possible for cases, and from a random side breast for women without cancer as done by others [35]. It is common practice in more recent studies to measure breast density from the contralateral (unaffected) breast for cases to avoid including the tumour in dense tissue estimation [36-39]; however, in practice breast density readings taken from ipsilateral and contralateral breasts have very similar distributions and similar estimates of breast cancer risk prediction [40], and *Cumulus* measures were highly correlated between breasts within this dataset [21]. Further, for this study we use the same mammogram for *Cumulus* and *AutoDensity* readings and so if there was bias in the measurement of exposure (breast density) it would affect both measures equally. The measurements were taken by one of four readers (a radiologist, a radiology registrar, and two research assistants), with high inter- and intra-reader reliability [21].

### Automated measurement of breast density

We developed an executable program (‘*AutoDensity*’) which automatically identifies the breast area in the mammogram (breast segmentation) and then classifies breast density in a similar way to *Cumulus* by identifying distinctly white tissue to be classified as ‘dense’ (breast density segmentation).

**Figure 1:**
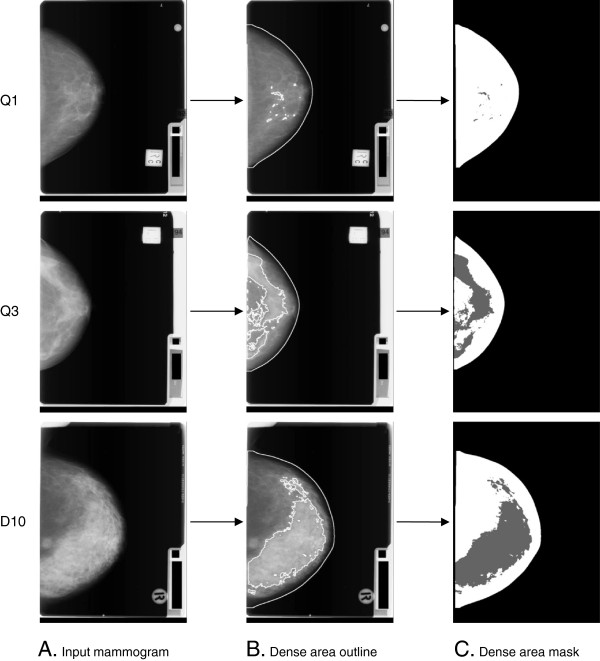
**Examples of the *AutoDensity *breast density segmentation process on three cranio-caudal-view digitised film mammograms.** Examples shown are from three women with different breast densities, from each of the lowest quintile (Q1), third quintile (Q3) and upper decile (D10) of the population distribution of dense area. For each woman, the breast density segmentation process is illustrated using **(A)** the input mammogram, **(B)** the dense area outline, and **(C)** the dense area mask.

Our approach to dense tissue segmentation does not require standardisation across images; the method finds an optimal threshold for each mammogram independently from any other mammogram in a dataset. We first improved the contrast and reduced the noise of individual images by smoothing the breast area with the median filter of radius one and then applying histogram contrast stretching [41]. Then, to automatically segment dense tissue within the breast area, we modified a recently published method by Kim *et al*. that had been developed on digital mammograms [42]. This method computes an optimal intensity threshold between dense and fatty tissues, which outlines the dense area on the breast as shown in Figure 1. Our methods are described in more detail in Additional file 1.

*AutoDensity* operates on Windows and Linux platforms and takes on average 2.9 seconds per image to produce measurements of breast density. *AutoDensity* outputs an indexed table of results along with images showing the original mammograms marked up with breast and breast density segmentations.

### Statistical analysis

Women were excluded from the analysis if they had any missing data on hormone therapy use at the time of screening, personal history of breast cancer, breast symptoms and family history of breast cancer. Women for whom *AutoDensity* could not produce breast density readings due to failures in the breast segmentation algorithm to outline the breast area were also excluded.

For all analyses we assessed dense area and percent density measures of breast density. These produced similar associations with outcomes. Evidence is mixed about whether dense area or percent density are stronger predictors of risk [43-45]: we report on dense area as done in our more recent publications [32,33] (results using percent density are available on request).

Statistical tests for interaction between screening round and breast density were performed for each screening outcome to determine whether the analyses should be conducted separately for first and subsequent screening rounds. There was no evidence of an interaction, therefore we categorised each breast density measure into whole-screened-population percentiles (weighting controls according to study sampling fractions to reproduce whole-population distributions). We then categorised breast density into the four lowest quintiles and the two upper deciles of their distributions (‘quintile-decile groups’) as done for our previous evaluations using *Cumulus*[32,33]*.* Age was categorised into groups 40 to 49, 50 to 54, 55 to 59, 60 to 64, 65 to 69 and 70 to 79 years.

We examined associations of *Cumulus* and *AutoDensity* with factors known to be associated with breast density (age, hormone therapy use, region of birth (Australia, Europe/North America, Asia and other), family history of breast cancer, breast symptoms, screening round and clinical outcome (small or large screen-detected cancer, interval cancer, no cancer)). We applied the Cuzick non-parametric test for trends across ordinal categories and Kruskal-Wallis non-parametric tests for differences between nominal groups. To illustrate the observed characteristics of tumours diagnosed in different breast density groups, we plotted histograms of the relative frequency of quintiles of tumour size according to breast density quintiles (using quintile categories due to inadequate power to assess by decile) and mode of detection (screen-detected or interval cancers).

To compare how well *Cumulus* or *AutoDensity* could be used to discriminate women’s clinical outcomes based on information from their screening mammograms, we generated receiver operating characteristic (ROC) curves and calculated the area under the curves (AUC). We repeated this exercise for small screen-detected cancers, large screen-detected cancers and interval cancers versus controls, and for interval cancers versus screen-detected cancers.

To assess the association between breast density and clinical outcomes, we conducted a range of analyses including several previously published from this dataset using *Cumulus* measures [21,32,33]. We used unconditional logistic regression to estimate odds ratios of all invasive breast cancers, small screen-detected, large screen-detected and interval cancers (versus no cancer) for quintile-decile groups of breast density. We also modelled the relative odds of an interval cancer (versus a screen-detected cancer) in order to estimate programme sensitivity. All models were adjusted for age, hormone therapy use, family history of breast cancer, symptoms and screening round. Since there was no evidence of an interaction between screening round and breast density, all results are presented for combined screening rounds.

As a secondary analysis, we compared *Cumulus* and *AutoDensity* measures by examining pairwise correlation coefficients, scatterplots of percentiles, Bland-Altman plots of agreement and quantile-quantile (Q-Q) plots of a 20% random sample of breast density values, and cross-classification tables of quintile-decile groups (Q-Q plots compare two distributions by plotting their quantiles against each other). All analyses were conducted in Stata 12.1 [46].

### Approvals and consent

This study was approved by the University of Melbourne Health Science Human Ethics Sub-Committee (Ethics ID 0932609) on 15 December 2009. All BreastScreen Victoria clients provide signed consent for use of their data for research purposes under the governance of the BreastScreen Victoria Board of Management, which approved this study on 22 September 2009.

## Results

We excluded 161 women (2%) in the study group who had missing questionnaire data. A further 247 women (4%) were excluded because *AutoDensity* failed to segment the breast area of their mammograms. Compared to successful *AutoDensity* reads, failed *AutoDensity* reads had higher *Cumulus* values for breast area (median 260,990 versus 204,084 pixels, *t* test *P* < 0.001) and dense area (29,376 versus 26,446 pixels, *t* test *P* = 0.08), and lower *Cumulus* values for percent density (13% versus 15%, *t* test *P* = 0.03). There were no failures in the breast density segmentation step.

The final sample for analysis included 6,327 women, comprising 2,818 women from first-round screening (411 small screen-detected cancers, 247 large screen-detected cancers, 174 interval cancers and 1,986 controls) and 3,509 women from subsequent round screening (242 small screen-detected cancers, 85 large screen-detected cancers, 193 interval cancers and 2,989 controls).

**Figure 2:**
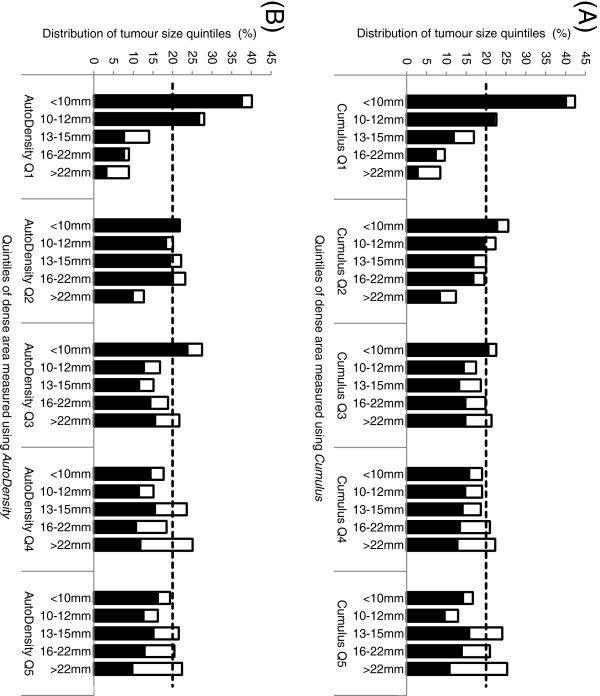
**Distribution of tumour size and mode of detection according to dense area quintiles measured using (A) *Cumulus *and (B) *AutoDensity*.** Screen-detected and interval cancers (marked as dark and light bars) are shown separately but stacked to indicate the size distribution of all cancers detected in each breast density quintile as well as the relative representation of screen-detected and interval cancers in this distribution. The dashed line represents the expected tumour size distribution within each breast density group if the distribution of tumour size did not vary according to breast density.

*Cumulus* and *AutoDensity* were similarly distributed according to age, hormone therapy use, region of birth, breast symptoms, and screening test outcome (Table 1). Histograms of tumour characteristics (size and mode of detection) according to breast density highlighted the shift from predominantly small screen-detected tumours in women with low breast density to larger screen-detected and interval cancers in women with high breast density, for both *Cumulus* and *AutoDensity* classifications of breast density (Figure 2).

**Table 1:** **Distribution of screened population percentiles of *Cumulus *** **and *AutoDensity *** **according to known breast density correlates**

**Variable**	**Categories**	**n (%)**	**Percentiles of dense area**	
			**Median (IQR)**^**#**^	
			*Cumulus*	*AutoDensity*
Age group (years)	40-49	581 (9%)	76 (53, 91)	69 (46, 86)
	50-54	1,201 (19%)	63 (36, 82)	60 (36, 81)
	55-59	1,282 (20%)	52 (27, 77)	51 (25, 75)
	60-64	1,214 (19%)	45 (23, 69)	44 (22, 70)
	65-69	1,192 (19%)	39 (19, 64)	41 (20, 69)
	70-79	857 (14%)	40 (18, 64)	41 (21, 69)
			z = -21.0, *P* < 0.001*	z = -16.0, *P* < 0.001*
Hormone therapy use at screening	No	4,909 (78%)	47 (23, 72)	47 (23, 73)
	Yes	1,418 (23%)	64 (37, 83)	62 (37, 81)
			*χ*^2^ = 179.9, p < 0.001^†^	*χ*^2^ = 127.0, p < 0.001^†^
Family history of breast cancer	No	6,111 (97%)	51 (25, 76)	50 (25, 75)
	Yes	216 (3%)	50 (27, 74)	56 (27, 76)
			*χ*^2^ = 0.2, *P* = 0.630^†^	*χ*^2^ = 1.4, *P* = 0.235^†^
Region of birth	Australia	4,109 (65%)	50 (25, 76)	51 (26, 76)
	Europe/North America	1,782 (28%)	51 (26, 75)	49 (23, 75)
	Asia	237 (4%)	58 (30, 78)	54 (40, 71)
	Other	198 (3%)	53 (27, 80)	52 (40, 71)
			*χ*^2^ = 4.7, *P* = 0.194^†^	*χ*^2^ = 4.8, *P* = 0.187^†^
Breast symptoms	None	5,964 (94%)	50 (25, 75)	50 (25, 75)
	No significant symptoms	363 (13%)	62 (35, 81)	59 (29, 79)
			*χ*^2^ = 20.4, *P* <0.001^†^	*χ*^2^ = 6.0, *P* = 0.014^†^
Screening round	First	2,818 (45%)	50 (25, 76)	51 (26, 77)
	Subsequent	3,509 (55%)	51 (26, 75)	50 (24, 75)
			*χ*^2^ = 0.1, *P* = 0.715^†^	*χ*^2^ = 3.6, *P* = 0.059^†^
Screening outcome	Small screen-detected cancers	653 (10%)	46 (23, 74)	47 (25, 78)
	Large screen-detected cancers	332 (5%)	58 (37, 79)	53 (36, 80)
	Interval cancers	367 (6%)	67 (44, 86)	70 (47, 88)
	No cancer (controls)	4,975 (79%)	49 (24, 74)	49 (23, 74)
			*χ*^2^ = 109.5, *P* < 0.001^†^	*χ*^2^ = 123.1, *P* < 0.001^†^

^#^A median value above 50 indicates that the subgroup has higher median breast density than the whole screened population (and vice versa); *Cuzick non-parametric test for trends across categories; ^†^Kruskal-Wallis non-parametric test for differences between groups. *IQR* interquartile range.

**Figure 3:**
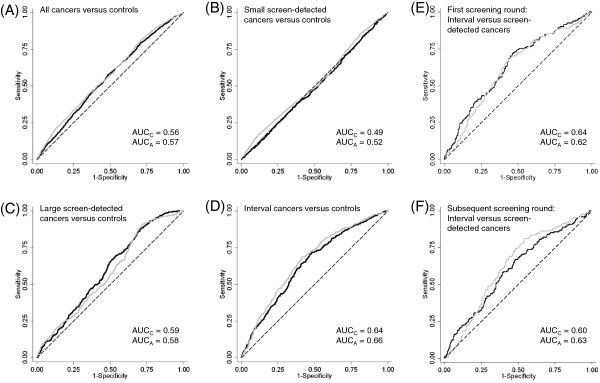
**Receiver operating characteristic (ROC) curves and area under the curve (AUC) values to assess the discriminatory performance of *Cumulus *and *AutoDensity *dense area.** ROC curves and AUC values are shown for various outcomes, including **(A)** all cancers versus controls, **(B)** small screen-detected cancers versus controls, **(C)** large screen-detected cancers versus controls, **(D)** interval cancers versus controls, **(E)** interval cancers versus screen-detected cancers for first round screening, and **(F)** interval cancers versus screen-detected cancers for subsequent round screening. *Cumulus* values are shown in black, *AutoDensity* values are shown in grey. The dashed line represents no predictive value.

ROC curves and their AUCs (Figure 3) showed that *Cumulus* and *AutoDensity* had a similar ability to discriminate between women who developed breast cancer and women who did not. Discrimination was strongest between women with interval cancers and controls (for example AUC = 0.66 for *AutoDensity*) and lowest between small screen-detected cancers and controls (for example AUC = 0.52 for *AutoDensity*).

**Figure 4:**
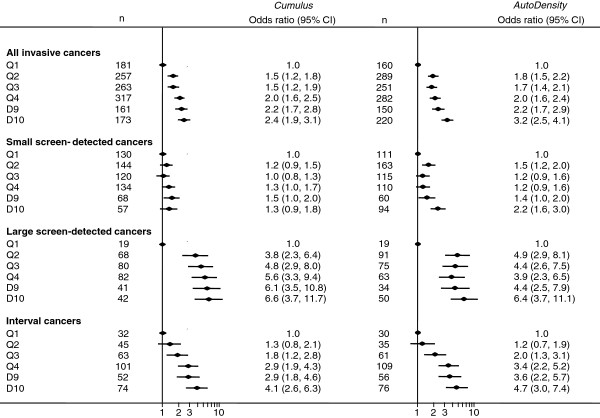
**Multivariate logistic regression of cancer risk and screening outcomes according to *Cumulus *and *AutoDensity*.** Breast density was measured as dense area and categorised into quintile-decile groups. Regression models were adjusted for age, hormone therapy use, family history of breast cancer, symptoms and screening round.

*Cumulus* and *AutoDensity* breast density measures showed a similar association with the odds of invasive breast cancers, large-screen detected cancers and interval cancers (Figure 4).

From models of odds ratios of interval versus screen-detected cancers, we predicted similar, graded associations between breast density and screening programme sensitivity for both *Cumulus* and *AutoDensity* (Table 2), showing that *AutoDensity* could differentiate expected programme sensitivity. For example, for women aged 50 to 54 years attending their first screening appointment with no symptoms, hormone therapy or strong family history of breast cancer, a woman in the lowest quintile of *AutoDensity* had an expected programme sensitivity of 76% (95% confidence interval (CI) 57% to 89%) whereas a woman in the highest decile of *AutoDensity* had an expected programme sensitivity of 68% (95% CI 46% to 84%).

**Table 2:** **Predicted programme sensitivity according to *Cumulus *** **and *AutoDensity***

		**Programme sensitivity (95% CI)**	
	**Dense area categories**	***Cumulus***	***AutoDensity***
First screening round	Q1	82 (62, 92)	76 (57, 89)
	Q2	82 (63, 92)	80 (61, 91)
	Q3	77 (57, 90)	76 (55, 89)
	Q4	73 (53, 87)	75 (54, 88)
	D9	74 (52, 88)	68 (46, 84)
	D10	65 (43, 83)	68 (46, 84)
Subsequent screening rounds	Q1	65 (41, 84)	59 (36, 79)
	Q2	66 (42, 83)	66 (42, 84)
	Q3	59 (36, 79)	58 (36, 78)
	Q4	54 (32, 74)	56 (34, 76)
	D9	54 (31, 76)	50 (29, 72)
	D10	44 (22, 67)	52 (30, 73)

Breast density was measured as dense area and categorised into quintile-decile groups. Predicted values are generated from regression modelling. We show the predicted programme sensitivity for 50- to 54-year-old women with no significant breast symptoms, not on hormone therapy and with no family history of breast cancer.

**Figure 5:**
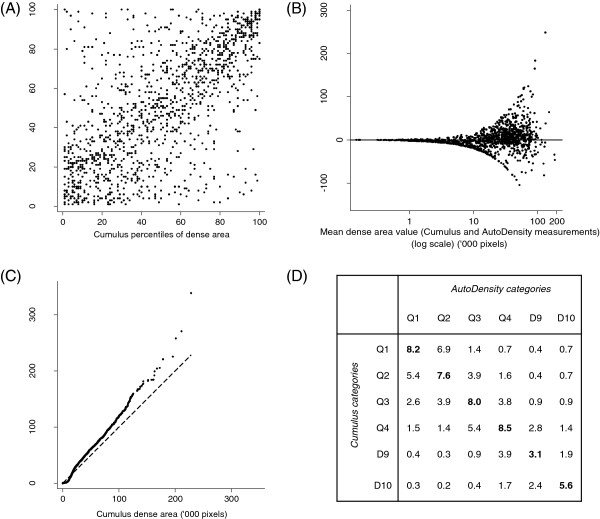
**Distribution and variation between *Cumulus *and *AutoDensity*. (A)** Scatter plot of screened population percentiles of dense area for a 20% random sample of the study group. **(B)** Bland-Altman plot of agreement between *Cumulus* and *AutoDensity* dense area for a 20% random sample of the study group. The x-axis shows the mean value of the *Cumulus* and *AutoDensity* measurements for each image, on a log scale. **(C)** Quantile-quantile plot of dense area percentiles of *Cumulus* against percentiles of *AutoDensity* (a deviation from the diagonal indicates a difference in distributions). **(D)** Cross-classification of quintile-decile groups (%).

There was a strong correlation between *Cumulus* and *AutoDensity* segmentation of the breast area (*r* = 0.98, *P* < 0.001), however the correlation between measures of breast density was modest (*r* = 0.63, *P* < 0.001), with discordant values for both low and high values of breast density (Figure 5a). *AutoDensity* dense area tended to be higher than *Cumulus* (median 21,293 versus 18,400 pixels), particularly for more dense breasts (Figure 5b and c). Using quintile-decile groups, 41% of *Cumulus* and *AutoDensity* breast density measurements were in perfect agreement and 40% were in near agreement (within one neighbouring category) (Figure 5d).

## Discussion

Automated breast density measurements from film screening mammograms using *AutoDensity* were comparable to (and often better than) *Cumulus* in terms of predicting breast cancer risk and key screening programme outcomes (Figure 4).

These findings suggest that *AutoDensity* is a valid tool for identifying groups of women at increased risk of breast cancer and at high risk of large screen-detected cancers or interval cancers, and therefore most likely to benefit most from more intensive screening modalities such as MRI, adjunctive ultrasound or shorter screening intervals. Quite low AUC values (Figure 3) show that using *AutoDensity* alone to classify individual women’s risk of various clinical outcomes is of limited benefit (as for *Cumulus*). As noted in a review paper by Vachon *et al*. [47], efforts to incorporate breast density into existing breast cancer risk models modestly improve classification of women’s risk but the models remain better suited to identifying risk groups for targeted health services, rather than providing absolute risk estimates to individuals. Breast segmentation was highly correlated between *Cumulus* and *AutoDensity* measures (*r* = 0.98, *P* < 0.001), showing that *AutoDensity* can successfully delineate the breast area of interest even in images with complex background features.

As a limitation, automated breast segmentation failed for 4% of mammograms in the dataset; this tended to occur when breast images overlapped with background artefacts or the image border, which was more likely with larger breasts. We are continuing to develop our algorithm to reduce this failure rate.

The correlation between *AutoDensity* measures and *Cumulus* was moderate (*r* = 0.63, *P* < 0.001), despite each measure showing a similar distribution according to age, hormone therapy use, region of birth, family history of breast cancer, breast symptoms at screening and clinical outcomes (Table 1 and Figure 2). This finding does not reduce the validity of our primary finding that both measures predict breast cancer risk and breast cancer screening outcomes with a similar effect. While *Cumulus* is in common use, it is not the gold standard measure of breast density: both measures have error in terms of describing the breast composition of dense and fatty tissue. Indeed, further investigation of visual features that produced non-correlated measures may enable further improvement of *AutoDensity*; this will be the focus of future work.

This study has a number of strengths. We used a large, unique dataset from a well-organised population screening programme that includes interval cancers identified through linkage to the cancer registry and good information on tumour size as well as personal characteristics of screening participants, which enabled us to adjust our models for potential confounders. We assessed an automated method that does not require modifications at the time of image capture as required by some other methods, such as the collection of pre-processed (raw) images from mammography machines [29,31] or the use of step wedges [27,30,31]. *AutoDensity* is not limited to specific machine brands and models of analogue mammogram machines because it flexibly identifies and removes the range of background artefacts generated by different film cassettes, and it replicates the *Cumulus* reader-driven approach of classifying as dense tissue the relatively bright components of the breast image. While *Cumulus* requires trained readers, is time-consuming and would have limited reliability in clinical practice, *AutoDensity* automatically completes readings in an average of 2.9 seconds, with perfect between-reader reliability (by definition, since it is fully automated). The program generates simple data tables of measurements and output images showing how the program has outlined the breast and the dense tissue.

The study did not include body mass index (BMI), which may modify the association between breast density measures and breast cancer risk [48]; our dataset was limited to information routinely collected by the BreastScreen programme, which does not include BMI; however, the primary aim of this study was to compare effect estimates from *AutoDensity* and *Cumulus* in the same models; and any bias in the model arising from excluding BMI should be the same for both breast density measures.

Our current analysis was confined to film mammograms taken during the period 1994 to 1996, utilising a large research dataset with existing *Cumulus* readings. *AutoDensity* is likely to be useful for research studies that require breast density estimation from older film mammograms, such as studies of long-term breast cancer risk. However, many screening services have adopted or will soon migrate to digital mammography. The breast density segmentation method implemented in this study was originally developed on digital mammograms and so it will need little modification for digital mammography; future work will aim to validate *AutoDensity* on data from BreastScreen Australia digital mammography services.

## Conclusions

This study demonstrates that, despite only modest correlation with *Cumulus* measures, automated measurement of breast density from digitised screening mammograms using *AutoDensity* performs similarly to *Cumulus* in terms of helping to identify groups of screening participants known to be at higher risk of breast cancer, interval and large screen-detected cancers, lower programme sensitivity, and larger tumour size at diagnosis. *AutoDensity* is a fast, stand-alone Windows or Linux program that is a validated breast density measurement tool suitable for digitised film mammograms.

## Abbreviations

AUC: Area under the curve; BI-RADS: Breast imaging reporting and data system™; BMI: Body mass index; CC: Cranio-caudal; CI: Confidence interval; DCIS: Ductal carcinoma *in situ*; MRI: Magnetic resonance imaging; Q-Q plots: Quantile-quantile plots; ROC: Receiver operating characteristic.

## Competing interests

Commonwealth Scientific and Industrial Research Organisation (CSIRO) has financially supported this study by contributing staff time for software development and manuscript writing. *AutoDensity* software may be commercialised in the future for use in research and clinical settings, with any financial gains to be shared equally between CSIRO and the University of Melbourne.

## Authors’ contributions

CN conceived of and coordinated the study. AK originally collected the images for the study and led *Cumulus* measurement of those images. YA, TE, MB and ML developed the image processing algorithms. ZA and ML implemented the image processing and managed image and clinical data. ZA and CN performed the statistical analysis and DE and AK advised on analytic methods. CN, ZA, DE, YA and AK drafted the manuscript. All authors read and approved the final manuscript.

## Supplementary Material

Additional file 1 Additional materials. Click here for file
