# Supplementary material for: AutoDensity: an automated method to measure mammographic breast density that predicts breast cancer risk and screening outcomes
Source: Breast Cancer Res. 2013 Sep 11;15(5):R80. doi: 10.1186/bcr3474 (PMC3978575; doi:10.1186/bcr3474)
Supplement: Additional file 1 — Additional materials. [file bcr3474-S1.docx]

# ADDITIONAL MATERIALS

***Breast segmentation***

**Figure A1** **Flow chart of the algorithm used to segment the breast from the image background.** As per convention, different shapes in the flow chart denote the following: input/output data (parallelogram), process (rectangle), or compound process or subroutine (rectangle with double sides).


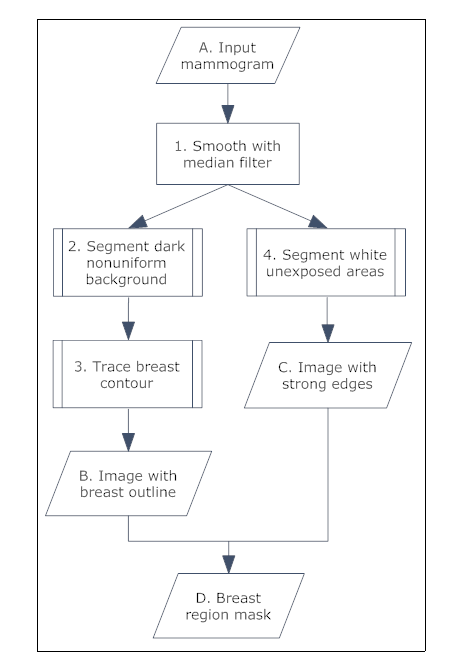


**Figure A2** **An example of the steps in the** ***AutoDensity*** **breast segmentation algorithm on a CC view digitised film mammogram.** Image caption labels (**A**, **B**, **C**, **D**) correspond to the labels in the flow chart in Figure 1.


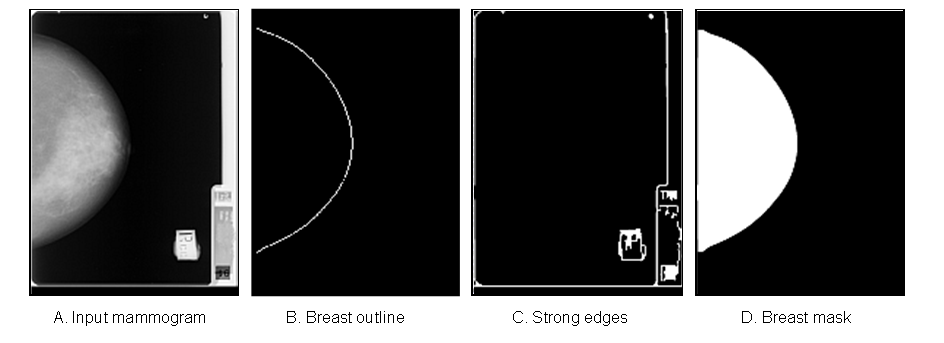


Breast segmentation followed the flow chart shown in Figure A1; an example is shown in Figure A2. To automatically segment the breast region, mammograms were smoothed with the median filter of radius one (step 1) and then a gradient magnitude filter with subsequent thresholding applied to obtain an image of components with homogeneous intensities (step 2). These components were formed by pixels whose intensities were smaller than a very low threshold value and the dark image background was the largest connected component in this image. Assuming that the breast region was to be found on the left side of the mammogram, the breast skin line was traced along the background on that side, and the top and bottom breast corners were identified (step 3). Finding the breast corners (where the breast intersects with the left edge of the x-ray film) was crucial for separating the breast area from artefacts such as vertical stripes of non-homogeneous background that can lie above and below the breast region and adjacent to the image edge.

In a parallel process following smoothing, we obtained an image of strong edges by thresholding the smoothed input image filtered with the gradient magnitude filter (step 4). As opposed to thresholding in step 2, in order to construct edges we selected pixels which intensities were higher than a large threshold value. In a digitised mammogram, strong edges could be formed, for example, by white unexposed areas or by image artefacts that present a high contrast in intensities with surroundings. Finally, the breast profile was smoothed and its corners were connected by horizontal lines to either an image edge or an edge created by white unexposed areas. The resulting shape represented the mask of the breast area. During segmentation, images where nametags and other tags intersected with the breast were identified and excluded from further analysis. It was done automatically by checking a number of conditions on the size and extension of the image background and breast skin line (in steps 2 and 3). Such segmentation failures affected approximately 4% of our image dataset.

***Breast density segmentation***

The breast density segmentation method published by Kim *et al*. computes an optimal intensity threshold between dense and fatty tissues to define dense and non-dense regions. The optimal threshold is found by combining two image features computed at each intensity level *t*. A statistical feature *D(t)* is a quadratic mean of standard deviations of image intensities in dense and fatty regions defined by *t*. The smaller the value of *D*, the better the two tissue types are separated by the threshold *t*. An edge magnitude feature *E(t)* is computed from the gradient magnitude of the input image and defines an edge strength along the threshold boundary. Since boundaries between the dense and fatty portions of the breast usually have high values of gradient magnitude, large values of *E(t)* correspond to intensity levels that best divide fatty and dense regions. Hence, the optimal threshold *t^i^_opt_* is found by maximizing the ratio *S(t)=E(t)/D(t)*, as described in detail by Kim *et al.* [1].

We have found that applying Kim’s method to digitised films as published often produces a false optimal threshold that separates a narrow band of low intensities along the breast skin line from the rest of the breast. Therefore, we modified the method to converge to an intensity level that separates dense from fatty tissues on digitised mammograms. In brief, our modification excludes outer parts of the breast projection from the evaluation of *S(t)*. This is achieved by performing an iterative search for the optimal threshold while decreasing a region of interest within the breast until a stopping condition is met. To decrease the region of interest, the breast mask is eroded with a sphere of increasing radius at each iteration, and the ratio *S(t)* is evaluated from the portion of the breast that lies inside an eroded breast mask. At each iteration *i*, an estimated optimal threshold *t^i^_opt_* is applied to compute *percent density* *PD_i_* over the whole breast projection. If *PD_i_* is larger than a predefined *PD_max_*, the algorithm proceeds to the next iteration unless the maximum number of iterations is reached or the size of an eroded breast mask becomes too small.

Prior to computing the optimal threshold, input mammograms were pre-processed by smoothing with the median filter of radius one, followed by histogram contrast stretching. We experimentally selected *PD_max_*=50%. It is possible to obtain a final value of *percent density* larger than *PD_max_* when one of the other two stopping conditions is met prior to the *percent density* upper limit condition. In our implementation, the maximum number of iterations is set to 20. The eroded breast mask size condition requires that the radius of a structuring element used in eroding the mask should not exceed 0.3 times the length of a breast mask bounding box in a current iteration.

*AutoDensity* was implemented in C++ using the ITK open-source library of software tools for image analysis [2]. The running time of the breast density segmentation algorithm averaged 0.6 seconds (maximum under 5 seconds) per 676x926 image. The average time for both breast segmentation and breast density segmentation was 2.9 seconds.

The algorithm was executed on a 2.4 GHz PC with Intel dual core and 2 GB of RAM running under Windows XP Professional operating system. No code optimization was performed.

**REFERENCES**

1. Kim Y, Kim C, Kim J-H: **Automated estimation of breast density on mammogram using combined information of histogram statistics and boundary gradients**. *Proc of SPIE Medical Imaging* 2010, **7624**:76242F.

2. Ibanez L, Schroeder W, Ng L, Cates J: **The ITK Software Guide**. In*.*: Kitware, Inc; 2005.
